# Extensive small cell lung cancer treated by integrated traditional Chinese and Western medicine: A case report

**DOI:** 10.1097/MD.0000000000041291

**Published:** 2025-01-17

**Authors:** Yuanqing Yang, Si Li

**Affiliations:** a Department of Acupuncture and Moxibustion, Tianjin Academy of Traditional Chinese Medicine Affiliated Hospital, Tianjin, P.R. China.

**Keywords:** case report, extensive stage, integrated traditional Chinese and Western medicine, small cell lung cancer

## Abstract

**Rationale:**

Patients with extensive small cell lung cancer (SCLC) generally have a dismal survival rate and are conventionally treated with chemotherapy. This study aimed to explore an alternative treatment approach by combining traditional Chinese medicine (TCM) with radiotherapy and chemotherapy.

**Patient concerns:**

A 68-year-old male was diagnosed with extensive-stage SCLC. Positron emission tomography/computed tomography scans indicated a high metabolism mass in the left hilar region, along with multiple lymph node metastases and metastatic tumors in both lungs. A left lung biopsy verified small cell carcinoma.

**Diagnoses:**

The patient was confirmed to have extensive stage SCLC based on imaging and biopsy results.

**Interventions:**

The patient received a comprehensive treatment regimen. TCM herbal prescriptions such as Qianjin Weijing Decoction, Wuwei Xiaodu Decoction, Xiaoxianxiong Decoction, and Xiaochengqi Decoction were administered. In parallel, standard Western medicine therapies, including chemotherapy (intravenous cisplatin and etoposide) and radiotherapy, were carried out. Oral ondansetron was given to alleviate nausea and vomiting caused by chemotherapy and radiotherapy.

**Outcomes:**

After 30 months, follow-up positron emission tomography/computed tomography demonstrated the complete disappearance of all cancerous lesions. The patient regained normal daily activities and experienced a substantially enhanced quality of life. Four years after the initial treatment, there has been no recurrence, and the patient persists in taking the original TCM decoction.

**Lessons:**

The combination of TCM and Western medicine in treating SCLC proves effective in managing systemic symptoms, mitigating bone marrow suppression, gastrointestinal reactions, immunosuppression, and other chemotherapy-induced adverse reactions. It also significantly prolongs the survival period, suggesting it as a preferable treatment option for SCLC.

## 1. Introduction

Lung cancer has become the most mortal among the malignant tumors, of which small cell lung cancer (SCLC) accounts for 14%.^[1–4]^ SCLC has a fast doubling speed and can lead to distant metastasis at the early stage. Most SCLC patients had blood metastasis at the initial diagnosis, and only 1/3 of the patients were at the limited stage. Although SCLC is highly sensitive to chemotherapy, it is prone to relapse and metastasis. The survival of patients with extensive stage SCLC is low and combined chemotherapy has become the main treatment. However, chemical drugs kill both tumor and normal human cells, so many patients fail to carry out standardized treatment on time as they cannot tolerate the toxic and side effects of chemotherapy. Nowadays, it is advocated to treat lung cancer with multiple methods and individualized treatments.^[5]^ Traditional Chinese medicine (TCM) can effectively improve the effect of radiotherapy and chemotherapy, prolong the survival period, reduce the side effects, and improve the quality of life for lung cancer patients.^[6]^ We combined disease differentiation and syndrome differentiation to treat a case of extensive stage SCLC, yielding a remarkable result. Therefore, we advocate the combination of modern medical knowledge and scientific and technological means based on syndrome and treatment differentiation of TCM and adjustment of medication rules, so as to provide some ideas for the time to introduce TCM in tumor treatment.

## 2. Case presentation

**Figure 1.**
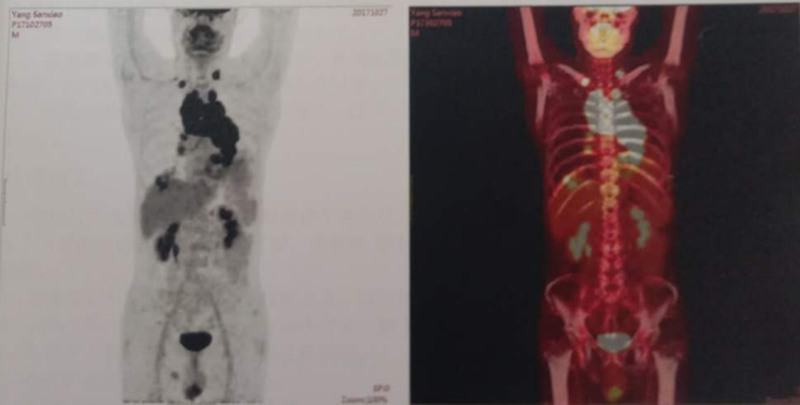
PET/CT (p17102705). PET/CT (p17102705) of Baotou Central Hospital showed hypermetabolic mass in left hilar area, which was considered to be malignant with multiple lymph node metastases in left supraclavicular region, right subclavian region, uppermost mediastinum, paratracheal, anterior vena cava, retrotracheal vena cava, paraaortic arch, aortic window, subcarina, and bilateral hilar regions, and metastatic tumor in both lungs. The left lung biopsy showed small cell carcinoma.

**Figure 2.**
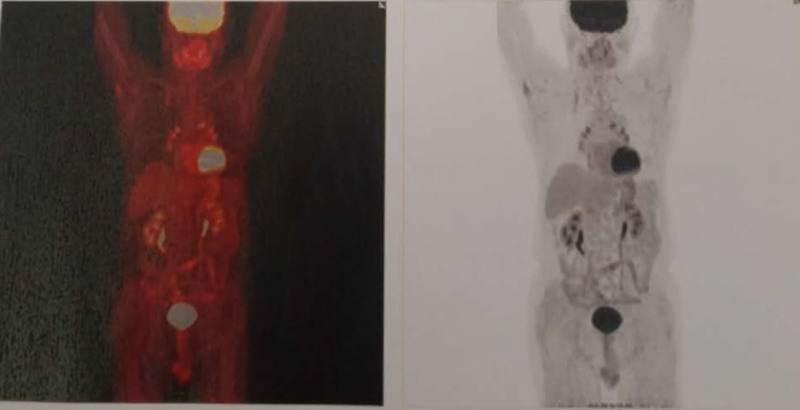
PET/CT (p17102705-2). The mass of hypermetabolic soft tissue in the left hilar region disappeared compared with the 2017.10 PET/CT result; the hypermetabolic enlarged lymph nodes in the original mediastinum and left hilar almost disappeared; there were multiple slightly hypermetabolic lymph nodes in the mediastinum and right hilum, some of which were accompanied with calcium deposition and possibly due to inflammatory proliferation, so regular follow-up checks were required; the left intrapulmonary metastasis disappeared; no abnormal uptake of imaging agents was found in the right 10th rib and left occipital lobe, though regular check was still required.

The patient was a 68-year-old man, who had his initial diagnosis on October 30, 2017. Before this, there was no family history of genetic diseases or medical treatment history.When the patient was hospitalized at a local county hospital for cough, shortness of breath, and fatigue. Chest CT showed (ID: 1710240055) left hilar mass and left upper lobe nodule shadow, so central pulmonary CA with intrapulmonary metastasis was considered. PET/CT (p17102705) (Fig. 1) of Baotou Central Hospital showed hypermetabolic mass in left hilar area, which was considered to be malignant with multiple lymph node metastases in left supraclavicular region, right subclavian region, uppermost mediastinum, paratracheal, anterior vena cava, retrotracheal vena cava, paraaortic arch, aortic window, subcarina, and bilateral hilar regions, and metastatic tumor in both lungs. The left lung biopsy showed small cell carcinoma. The first cycle of chemotherapy started on October 28, 2017, and the drug used was cisplatin + etoposide. The patient was in his first cycle of chemotherapy when he visited our team, showing symptoms of yellow sticky phlegm, shortness of breath, lack of appetite, weight loss, poor sleep, 1 dry stool per day, normal urination, dark red tongue, yellow greasy coating, and stringy, slippery, rapid pulse. The treatment aimed at clearing away heat and detoxification, diffusing lung and relieving cough, and resolving hard lumps. Qianjin Weijin Decoction, Wuwei Xiaodu Decoction, and Xiaochengqi Decoction were prescribed as follows: 50 g of *Hedyotis diffusa*, 50 g of *Scutellaria barbata*, 30 g of *mulberry leaf*, 20 g of *honeysuckle*, 25 g of *Chrysanthemum indicum*, 25 g of *dandelion*, 25 g of *Scutellaria baicalensis*, 50 g of *reed root*, 20 g of *walnut kernel*, 25 g of wax gourd seed, 30 g of *raw coix seed*, 15 g of *rhubarb*, 20 g of *Fructus aurantii Immaturus*, 20 g of *Magnolia officinalis*, 25 g of *Solanum nigrum*, 30 g of *Radix asparagi*, 30 g of *Ophiopogon japonicus*, 50 g of *Trichosanthes kirilowii*, 20 g of *Fritillaria thunbergii*, 30 g of *Rehmannia glutinosa*, 25 g of *Peony bark*, 50 g of *Prunella vulgaris*, 10 g of *Coptis chinensis*, 20 g of *Gardenia jasminoides*, 20 g of *Apricot kernel*, 20 g of Radix stemonae, 30 g of *Figwort*, and 30 g of *Ranunculus ternatus*. There were 28 doses in total, which were decocted in water and taken orally once a day. The second hospital visit happened on November 29, 2011. The patient finished the second cycle of chemotherapy on November 28, 2011, and the medicine was the same as before. Meantime, lung radiotherapy was conducted for 30 times. During this period, the whole body bone scan showed no abnormality, and the lung lesions were smaller than before. Blood routine examination showed leucopenia, so subcutaneous injection of granulocyte growth factors and orally taken Leucogen tablets were prescribed. The patient had symptoms of relieved cough, hair loss, and poor appetite, and did not complain of other obvious discomforts. The stool was thin and occurred 3 to 5 times a day, and the urine was normal. The tongue was pale, the coating was white and greasy, and the pulse were stringy and slippery. So, *Scrophularia* was removed from the original prescription, and 60 g of *Caulis Spatholobus* and 30 g of *Rhizoma Paridis* were added. The patient was told to stick to this decoction for 3 months, 1 dose per day. The fourth chemotherapy was completed on February 14, 2018. Chest CT examination in Baotou Central Hospital showed space-occupying lesion in left hilum, multiple lymphadenectasis in mediastinum, though the lesion was smaller than before. Also, exudative lesion in upper lobe of left lung and emphysema were present. Due to serious bone marrow transplantation and gastrointestinal tract reactions, the patient was suggested to reexamine after 3 months. The third visit was on March 1, 2018, and the patient showed symptoms such as dry mouth, fatigue, frequent defecation, occasional fever at night, dark red tongue, white coating, and stringy, slippery, rapid pulse. He complained no other obvious discomforts and had good appetite and sleep. Considering these, *Gardenia jasminoides* was removed from the previous prescription and the amount of *rhubarb* was adjusted to 5 g while 30 g of *Bupleurum* was added. Again, there were 28 doses in total and 1 dose per day was prescribed. Afterwards, the patient paid repeated visits. When his fever symptom disappeared, bupleurum was removed from the recipe and the previous prescription continued. On November 17, 2018, enhanced cranial MRI revealed a quasi-circular intense DWI signal in his left occipital lobe, which was possibly metastasis. At the follow-up visit after 30 times of local radiotherapy, the patient described symptoms like occasional headache, no fever, and no other discomforts. He had a dark red tongue, white tongue coating, and stringy, slippery pulse. According to the symptoms and signs of the patient, 20 g of *Ligusticum wallichii* was added in the previous prescription. The patient continued to take this prescription, with 1 dose per day. 2 months later, the lesions disappeared as shown in enhanced head MRI. In August 2019, bone scan showed increased salt metabolism in the right 10th rib, which could be bone metastasis. After that, local radiotherapy and first-line chemotherapy (cisplatin + etoposide) were performed for 4 cycles. At a follow-up visit during the period, the prescription remained unchanged except that 30 g of *Corydalis* was added. Next, enhanced chest and whole abdomen CT and enhanced head MRI were performed every 3 months, which showed no disease progression. The patient’s condition was stable in the follow-up visits and treated accordingly. As shown in the PET/CT (P17102705-2) scan performed on May 28, 2020 (Fig. 2), the mass of hypermetabolic soft tissue in the left hilar region disappeared compared with the 2017.10 PET/CT (p17102705) result; the hypermetabolic enlarged lymph nodes in the original mediastinum and left hilar almost disappeared; there were multiple slightly hypermetabolic lymph nodes in the mediastinum and right hilum, some of which were accompanied with calcium deposition and possibly due to inflammatory proliferation, so regular follow-up checks were required; the left intrapulmonary metastasis disappeared; no abnormal uptake of imaging agents was found in the right 10th rib and left occipital lobe, though regular check was still required. Through the systematic integrated treatment of traditional Chinese and Western medicine, which included cisplatin, etoposide, and ondansetron, the side effects of chemotherapy, such as bone marrow suppression, gastrointestinal reactions and immunosuppression, were effectively controlled. Ultimately, the cancerous lesions disappeared. The patient felt clear-minded, energetic, and had good appetite, decent sleep, and normal urination and defecation. His weight was significantly increased, his daily living and working was no longer impeded, and the quality of life was obviously improved. After 4 years of follow-up, no abnormality has been found.

## 3. Discussion

Lung cancer has the highest incidence and mortality among malignant tumors, which seriously threatens human health and life. Every year, over 1 million new cases of lung cancer occur worldwide, of which SCLC accounts for 15 to 20%.^[7]^ SCLC can be divided into limited stage and extensive stage ones. Extensive stage SCLC accounts for 60 to 70% of all SCLC and most cases have reached stage III–IV at the time of diagnosis. The 5-year survival of patients with extensive stage SCLC is <10%, and combined chemotherapy has become the main treatment. Therefore, how to improve the efficiency of chemotherapy, prolong the survival time, reduce the side effects, and improve the patient’s quality of life have become the main problems in treating extensive stage SCLC.^[8]^ Dr Li has inherited his family’s knowledge and accumulated rich experience in the treatment of various types of cancer with TCM as adjuvant therapy to adiotherapy and chemotherapy, which has achieved better curative effects. In this case, at the initial diagnosis, the patient had irritable cough, yellowish and sticky phlegm, dark red tongue, yellow and greasy coating, and stringy, slippery, and rapid pulse. Considering the phlegm and toxin accumulation in the lung, the patient was prescribed with large quantities of lung clearing, heat clearing, phlegm reducing, and detoxicating TCM. The treatment aimed at clearing away heat and detoxification, clearing lung and reducing phlegm, and resolving hard lumps. Qianjin Weijin Decoction, Wuwei Xiaodu Decoction, Xiaoxianxiong Decoction, and Xiaochengqi Decoction were included and modified accordingly. In Xiaoxianxiong Decoction, *Trichosanthes* is used to relax chest, relieve lumps, clear lung, and remove phlegm, whereas *Coptis chinensis* specializes in clearing heart and relieving heat, supplemented by *Gardenia jasminoides* for removing the source of heat. In Qianjin Weijin Decoction, the reed root and wax gourd seed are used to clear lung and reduce phlegm. Besides, in combination with *Trichosanthes kirilowii*, the goal of clearing lung, reducing phlegm, relaxing chest, and resolving lumps can be achieved. In Wuwei Xiaodu Decoction, *honeysuckle* and *Chrysanthemum indicum* are good at clearing heat, detoxification, and resolving lumps. *Honeysuckle* functions on the lung and stomach, which can relieve the heat toxin at middle and upper jiaos. *Chrysanthemum indicum* functions on the liver channel to clear the heat of liver and gallbladder. The 2 drugs combined are good at clearing Qi and dividing heat concentrations. Dandelion is used to clear away heat and toxin, and eliminate the dampness and heat of lower jiao. *Begonia fimbristipula Hance* is replaced by *Solanum nigrum* to clear away heat, detoxicate, resolve lumps, and activate blood circulation. The combination of the 5 herbs can clear both Qi and blood, treat the 3 jiaos simultaneously, and dividing the heat concentrations of the 3 jiaos. Xiaochengqi Decoction was taken to bring medicine downward, give pathogenic factors an outlet, and discharge turbidity toxin by excretion. In it, *Hedyotis diffusa*, Radix stemonae, and *Fritillaria thunbergii* can clear lung, reduce phlegm, and relieve cough; Radix asparagi, *Radix Ophiopogonis*, and *Radix Scrophulariae* are used to nourish yin, clear and moisten lung, which rapidly purge to preserve yin, eliminate pathogenic factors without hurting health; raw *Rehmannia glutinosa* and peony bark are used to clear away heat and cool blood. Tumors are often caused by interacting phlegm stasis and turbidity toxin. In this case, this cause was possibly revealed by the patient’s dark tongue. As a result, walnut kernel, *Scutellaria barbata*, and *Ranunculus ternatus* are used to regulate Qi, activate blood circulation, reduce stasis, and resolve lumps. Chronic diseases tend to consume yin blood inwardly, when combined with radiotherapy and chemotherapy, bone marrow suppression can be obvious. At the second hospital visit, Caulis Spatholobus and Paris polyphylla were added in the prescription. *Caulis Spatholobi* is bitter, sweet, and warm, which functions on blood and is good at nourishing blood, warming channels, and activating collaterals. *Paris polyphylla* promotes the power of clearing. At the third visit, the patient had a dry mouth and occasional fever at night. So *Bupleurum* in the recipe of Xiaochaihu Decoction was added to clear away the heat. The follow-up checks revealed stable conditions for the patient, so the original prescription was used. Another check during the period showed brain metastasis, so *Ligusticum wallichii* was added to promote Qi, activate blood circulation, relieve pain, and carry other drugs upward. At the later stage, the patient had obvious rib pain, and bone scan showed bone metastasis. Therefore, *Corydalis* was added in the original prescription.*Corydalis* functions on the liver channel and is good at promoting Qi and relieving pain, which can also lead medicine inward. The whole prescription also has the clearing and nourishing effect of *Baihe Gujin* Decoction and the clearing effect of *Shengdi Danpi* Decoction. In this case, the combination of various drugs had the effect of clearing away heat and toxin, clearing lung and reducing phlegm, and resolving hard lumps, thereby removing the disease.

## 4. Conclusion

In this case, disease differentiation and syndrome differentiation were combined to treat extensive stage SCLC. Although it is a single case, it has demonstrated the advantages of integrated traditional Chinese and Western medicine in the treatment of lung cancer. According to modern pharmacological research, TCM has the multicomponent, multi-target, multichannel collaborative regulatory effect, in which there are many active monomer components and complex and diverse mechanisms. TCM plays a variety of curative effects in stages such as inhibiting tumor cell proliferation and cell invasion and metastasis, promoting tumor cell apoptosis, regulating tumor microenvironment and antitumor angiogenesis, and reversing drug resistance.^[9,10]^ On the basis of syndrome and treatment differentiation by TCM and adjustment of drug use, combined with modern medical knowledge and scientific and technological means, mutual complementarity of traditional Chinese and Western medicine and close combination of clinical and basic research can be realized, so as to provide further basis for tumor treatment. In modern cancer research and treatment, even if the tumor cannot be cured, “survival with tumor” is advocated that aims to improve the patient’s quality of life. This is exactly where TCM can intervene. How to better achieve multidisciplinary combination in the treatment still needs more scientific, rigorous, large-scale clinical research. The opportunity of TCM intervention should be grasped to highlight the charm of integrated traditional Chinese and Western medicine in the treatment of malignant tumors.^[11]^ This research is only a report of a typical case and lacks a large-sample randomized controlled trial to provide high-quality evidence. However, the successful case of this medical record will surely serve as the direction for future research and development.

## Acknowledgments

The authors thank Dr Weishi Li for the enormous help in the treatment.

## Author contributions

**Writing – original draft:** Yuanqing Yang, Si Li.

**Writing – review & editing:** Yuanqing Yang, Si Li.
